# Genomic and Biomarker Innovations in Predicting Kidney Transplant Rejection

**DOI:** 10.3390/jcm14113642

**Published:** 2025-05-22

**Authors:** Rachana Punukollu, Sandesh Parajuli, Harshad Chaudhari, Girish Mour

**Affiliations:** 1 Division of Nephrology, Mayo Clinic, Phoenix, AZ 85054, USA; punukollu.rachana@mayo.edu (R.P.);; 2 Division of Nephrology, Department of Medicine, University of Wisconsin School of Medicine and Public Health, Madison, WI 53705, USA

**Keywords:** kidney transplant rejection, biomarkers, donor-derived cell-free DNA, gene expression profile, subclinical rejection, exosomes, torque teno

## Abstract

Currently, approximately 90,000 patients are on the kidney transplant waitlist in the United States, including 10,000 individuals awaiting re-transplantation due to prior graft failure. Allograft rejection remains a leading cause of kidney transplant failure. While the current gold standard for diagnosing rejection is tissue biopsy, it is invasive and impractical for routine or longitudinal graft surveillance. This review summarizes the current landscape of non-invasive biomarkers for detecting and predicting kidney transplant rejection, with a focus on both historical context and recent advancements. In particular, we highlight the roles of donor-derived cell-free DNA (dd-cfDNA) and gene expression profiling (GEP) in identifying acute rejection. We also discuss emerging biomarkers such as torque teno virus (TTV), which has shown potential as an indirect indicator of immunosuppression levels and rejection risk. Importantly, this review excludes biomarker studies that rely on tissue biopsy, emphasizing non-invasive approaches to rejection monitoring.

## 1. Introduction

Kidney allograft failure is the fourth leading cause of end stage renal disease (ESRD) in the United States [1]. Allograft rejection has been postulated to be one of the major causes of allograft failure in kidney transplant (KT) recipients, resulting in thousands of graft failures every year worldwide [1]. However, with advances in modern immunosuppressive treatments, rejection rates have dropped from 50–60% in the 1980s to less than or equal to 9% in 2021 depending on age and use of lymphocyte-depleting agents [2,3], but with only modest improvements in long-term graft survival [4]. Despite these improvements, acute rejection (AR) has a significant impact on recipient mortality, morbidity, financial hardship, and quality of life [5] with associated risk of recurrent episodes of rejection, graft loss, and return to dialysis [6,7]. In addition, subclinical rejection, detected on a surveillance biopsy, is shown to be associated with poor short- and long-term graft outcomes [8].

The gold standard for rejection diagnosis remains kidney transplant biopsy. However, it is invasive, expensive, impractical for serial testing, prone to sampling and interpretation errors [9], and has an approximately 1% risk of major complications [10]. Other signs of graft dysfunction include rising serum creatinine, de novo rise in preformed donor-specific antibodies (DSAs), but lacking specificity and sensitivity [11], and delay in generation of levels [12].

As a result of these shortcomings, alternate non-invasive biomarkers that can predict and allow earlier detection of graft injury are needed. Novel biomarkers have shown higher precision in detecting subclinical rejection before the clinical manifestations arise, along with superior test characteristics like sensitivity, specificity, and negative predictive value (NPV) compared to current tests used in clinical practice [13,14,15,16,17]. Organ transplants involve the transfer of genetic material from a donor to the recipient [18]. Hence, genetic biomarkers have the potential to carry information on graft health.

In this narrative review, we would like to focus on non-invasive biomarkers available in predicting rejection in the field of kidney transplantation. Human leukocyte antigen (HLA) markers or DSA or biomarkers involving the tissue biopsy have not been reviewed in this article.

## 2. Materials and Methods

A narrative literature review was conducted using the PubMed database to identify articles relevant to biomarkers in kidney transplant rejection. The following keywords have been used for the literature search: ’biomarker’, ‘kidney transplant’, ‘donor derived cell free DNA’, ‘rejection’, ‘gene expression biomarkers’, ‘torque teno virus’, ‘immunogenic cells’, ‘transitional B cell cytokines’, ‘chemokines’, ‘mRNA-based signatures’, ‘exosomes’, ‘Q score’. Studies including kidney transplant recipients (KTRs) < 18 years old and multiorgan transplant recipients and studies only exploring the role of renal biopsy in detecting kidney transplant rejection were excluded.

## 3. Biomarkers in Rejection

### 3.1. Initial Discovery

The initial evidence of DNA microchimerism in solid organ transplantation (SOT) dates to the 1990s [19,20]. In 1998, Yo and colleagues were the first to describe the detection of Y chromosome genetic material in female KTRs of male organ donors [21]. The researchers proposed that donor-derived cell-free DNA (dd-cfDNA) was a marker of graft cell death and could be used as a marker to detect graft rejection.

In 1999, Zhang et al. [22] reported quantitative PCR (qPCR) to quantify genome equivalents (GEs) where 1 GE represents the quantity of a particular DNA sequence in 1 diploid male cell of urinary sex-determining region Y gene and β-globin gene sequences in 14 female KTRs with male donors, and found a median of 8.7% (range 1.9–26.4%) urinary dd-cfDNA. GEs were elevated during acute rejection episodes and returned to baseline levels following treatment. This is the first study to report assessment of cell-free DNA (cfDNA) in AR which was later confirmed in other studies.

In 2013 Sigdel et al. [23] reported digital PCR to inspect the role of dd-cfDNA in kidney transplant rejection by analyzing Y chromosome cfDNA in biopsy-matched urine samples of 21 pediatric female KTRs with male donors. The mean dd-cfDNA was significantly elevated in those with AR (20.5 ± 13.9) compared to stable grafts (2.4 ± 3.3) and those with chronic allograft injury (2.4 ± 2.4). However, the mean cfDNA levels were similar to BK nephropathy (20.3 ± 15.7), making it indistinguishable from AR [23]. These earlier studies used the Y chromosome to quantify cfDNA, limiting the test only to female transplant recipients with male donors.

### 3.2. Donor-Derived Cell-Free DNA (dd-cfDNA)

Cell-free DNA (cfDNA) consists of extracellular double-stranded DNA fragments released into the bloodstream from nucleosomes after cellular apoptosis or necrosis or by active secretion from living cells. The circulating cfDNA fragments are small in size, ranging from 120–200 base pairs depending on the cellular origin, with a median half-life of 30 min to 2 h [24,25]. The presence of cfDNA in bodily fluids is indicative of various physiological and pathological processes, making it a promising biomarker in multiple fields. In oncology, cfDNA has shown significant potential for cancer detection and monitoring [26,27,28]. In obstetric medicine, it serves as a non-invasive tool for prenatal detection of aneuploidies [29], and recently its application is expanding in solid organ transplantation.

Donor-derived cell-free DNA (dd-cfDNA), a subset of cfDNA, is released from donor graft cells during organ injury, such as rejection or in the setting of infection. Particularly, dd-cfDNA constitutes only a small portion of the total cfDNA found in the recipient’s blood. The levels of dd-cfDNA increase in the event of organ injury, i.e., rejection or infection. dd-cfDNA can be filtered through the glomerular filtration system and released into the urine [23]. The ability to detect dd-cfDNA in both urine and plasma increases its utility as a non-invasive biomarker for monitoring of allograft health in high-risk patients.

#### 3.2.1. Methods of dd-cfDNA Quantification

There are two methods of dd-cfDNA quantification: random and targeted techniques, and each has its own distinct advantages for detecting dd-cfDNA in transplant recipients. Random techniques use adapter ligation followed by next-generation sequencing (NGS). Targeted techniques usually involve the use of single-nucleotide polymorphisms (SNPs), digital droplet PCR (ddPCR), or quantitative PCR (qPCR). Developed by Beck and colleagues in 2013 [30], in ddPCR a single PCR sample is partitioned into 20,000 individual droplets. This partition of target molecules into droplets creates a highly precise quantification method without the use of standard curves. This method demonstrates superior precision to qPCR, especially when low levels of DNA are available [14,30,31]. ddPCR results are also known to have a shorter turnaround time of 1 day in contrast to NGS which can take 2–3 days [32].

A multicenter circulating donor-derived cell-free DNA in blood for diagnosing acute rejection in kidney transplant recipients (DART) study by Bloom et al. [15] prospectively analyzed 107 biopsy specimens from 102 KTRs, of which 27 had AR, including 11 acute T-cell-mediated rejections (TCMRs), 6 active antibody-mediated rejections (AAMR), and 10 chronic AAMRs (CAAMRs). The dd-cfDNA level discriminated between biopsy specimens showing any rejection (T-cell-mediated rejection or antibody-mediated rejection (AMR)) and controls (no rejection histologically), *p* < 0.001 (receiver operating). Positive and negative predictive values for active rejection at a cutoff of 1.0% dd-cfDNA were 61% and 84%, respectively. The AUC for discriminating AMR from samples without AMR was 0.87 (95% CI, 0.75 to 0.97). Positive and negative predictive values for AMR at a cutoff of 1.0% dd-cfDNA were 44% and 96%, respectively. Median dd-cfDNA was 2.9% (AMR), 1.2% (T-cell-mediated types ≥ IB), 0.2% (T-cell-mediated type IA), and 0.3% in controls (*p* = 0.05) for T-cell-mediated rejection types ≥IB versus controls.

#### 3.2.2. Current Studies on dd-cfDNA in Kidney Transplantation

Sigdel et al. [14] retrospectively evaluated 193 KTRs with 217 biopsies, of which there were 32 ARs, 72 with borderline rejection (BL), 82 stable allografts (STAs), and 25 were other injuries (OIs). Median dd-cfDNA was significantly higher, 2.32%, in the AR (*p* < 0.001) compared to non-AR groups, BL (0.58%), OI (0.67%), and STA (0.40%). At the 1% cutoff, the sensitivity was 89%, specificity 73%, and assuming a prevalence of 25%, positive predictive value (PPV) was 52% and NPV 95% with an AUC of 0.87 [14].

There have been more than 20 studies that have assessed cfDNA in urine or plasma through various methodologies in KTRs. Although the above studies have shown significant differences in dd-cfDNA levels between those with and without AR, a few studies have failed to validate these findings [33,34]. Three meta-analyses have investigated the role of dd-cfDNA as a non-invasive biomarker in diagnosing kidney transplant rejection [35,36,37]. In a recent meta-analysis, Xing et al. [36] analyzed nine articles focused on the diagnostic accuracy of dd-cfDNA for AR. The sensitivity, specificity, AUC, odds ratio (OR), positive likelihood ratio (PLR), and negative likelihood ratio (NLR) of dd-cfDNA were 0.59 (95% CI, 0.48–0.69), 0.83 (95% CI, 0.76–0.88), 0.80, 7 (95% CI, 5–10), 3.5 (95% CI, 2.8–4.5), and 0.49 (95% CI, 0.40–0.61), respectively. The biomarker was shown to have higher specificity, higher PLR, and lower NLR. The high NPV adds value by acting as an excellent ‘rule-out test’.

Oellerich et al. [38] utilized a ddPCR test (TheraSure-Transplant Monitor) and prospectively compared absolute (cp/mL) and fractional (%) dd-cfDNA levels in those with AR (n = 15) and without AR (n = 83). Absolute levels and fractional levels were 3.3-fold (82 copies/mL versus 25 copies/mL) and 2-fold higher (0.57% versus 0.29%), respectively, in those with AR compared to the stable patients. Absolute levels had higher AUC than fractions (0.83 versus 0.73, *p* = 0.02) when comparing AR with stable grafts. The fractional cfDNA is also prone to variability during events like inflammation illnesses, sepsis, exercise, etc. which can modify the proportion of dd-cfDNA in total cfDNA, but absolute cfDNA obviates this limitation.

#### 3.2.3. Commercial Assays

In the United States, there are three commercially available assays to measure dd-cfDNA in kidney transplant recipients (KTRs), *AlloSure* by CareDx, *Prospera* by Natera, and *TRAC* by Viracor Eurofins. AlloSure (CareDx, Brisbane, CA, USA), Prospera (Natera Inc., San Carlos, CA, USA) and TheraSure (commercially available in Germany, TheraSure-Transplant Monitor; Liquid Biopsy Center GmbH, Gottingen, Germany) use a targeted approach. AlloSure and Prospera use PCR-based NGS read-out, without the need for donor or recipient genotyping, while TheraSure is a PCR-based test with ddPCR read-out out which requires recipient genotyping [32]. TRAC (Eurofins Viracor, Inc., Lee’s Summit, MO, USA) uses a random approach and requires recipient genotyping.

AlloSure uses a panel of 405 SNPs that are associated across 22 somatic chromosomes, while Prospera utilizes 13,392 SNPs from 4 chromosomes. Both tests allow dd-cfDNA measurement in kidney transplant patients without requiring knowledge of donor genotypes [39]. All these tests require whole blood and are currently carried out in central laboratories. However, there is a move to develop kit-based testing which can be run out of central laboratories [40]. These tests have an acute rejection (AR) threshold of 0.69–1% and are highly predictive of antibody-mediated rejection (AMR) compared to T-cell-mediated rejection (TCMR) as shown in Table 1. Several studies have validated these dd-cfDNA tests against the current gold standard test, i.e., renal biopsy. AlloSure and Prospera are currently approved by the FDA. In TRAC plasma is extracted from the whole blood into Streck BCT tubes and the sample is analyzed for cfDNA within 7 days of collection. This test utilizes NGS and recipient genotype data to calculate the cfDNA percentage. TRAC has not been approved by the FDA for clinical use yet [41]. A small prospective study compared two currently available tests—AlloSure and Prospera—using a validated 1% cutoff to detect rejection. Prospera was shown to have higher sensitivity in detecting TCMR compared to AlloSure, although both the tests were correctly able to identify all the rejections when a 0.5% cutoff was used [42]. In a different study, AlloSure demonstrated a shorter sampling to result turnaround time compared to Prospera [43].

Current commercial assays (Table 1) have made it possible to differentiate donor DNA from recipient DNA, without the need for genotyping, regardless of sex. Several studies have validated currently available dd-cfDNA assays.

#### 3.2.4. Role in Predicting Types of Rejection

Multiple studies have shown superior predictability of the dd-cfDNA assay in detecting and differentiating AMR from TCMR and no rejection. Median dd-cfDNA levels are higher in AMR (1.35 to 2.9%) compared to TCMR (0.27 to 1.2%) and no AR (0.3–0.67%) [15,45]. In addition, this assay has also shown superior sensitivity, specificity, and NPV in differentiating AMR from no AMR [15,45,46,47]. dd-cfDNA was also shown to detect AMR regardless of the DSA status. In the recent Trifecta study [16], it was shown that mean dd-cfDNA levels were elevated in all AMR cases, both DSA-positive AMR (mean = 2.1%) and DSA-negative AMR (mean = 1.7%). dd-cfDNA was a better predictor of AMR than DSA (AUC 0.85 vs. 0.66, respectively), making it a superior biomarker to diagnose rejection [16].

Its role in detecting TCMR has been unclear. Some studies have shown higher median dd-cfDNA levels (>1%) with high-grade (IB) and active TCMR [15,16], whereas other studies have shown lower dd-cfDNA levels in TCMR, similar to those without rejection [45]. The false negative results in TCMR cases could be due to differences in assay quantification techniques to amplify DNA fragments to detect dd-cfDNA. TCMR involves interstitial inflammation and tubulitis leading to extensive degradation of dd-cfDNA resulting in the formation of smaller fragments contrary to what is seen in AMR [32]. Absolute dd-cfDNA quantification has well discriminated between any AR (AMR or TCMR), borderline rejection, and no rejection [48].

dd-cfDNA is a more sensitive marker to AMR than TCMR at validated thresholds. The underlying reason could be due to differences in cell degradation pathways. In AMR, complement-led recruitment of the membrane attack complex leads to cell lysis and release of more circulating cfDNA. In TCMR, phagocytosis after apoptosis can sequester most of the intracellular contents leading to less cfDNA [49]. dd-cfDNA levels may be elevated several days prior to rejection as diagnosed by biopsy. However, although this biomarker has potential to detect graft injury, its role is still being explored, especially in non-AMR settings and other forms of graft injury including infection, and the role of ongoing surveillance.

The Center for Medicaid and Medicare Service (CMS) issued a proposed local coverage determination (LCD) in August 2023, restricting coverage of non-invasive biomarker testing. However recently, CMS has retracted the proposed LCD and current coverage for non-invasive biomarkers remains unchanged [50].

#### 3.2.5. Advantages

1. Non-invasive biomarker highly sensitive for AMR.
2. High negative predictive value helps in avoiding unnecessary invasive diagnostic biopsy.
3. It has a short half-life, and levels rapidly decline after successful rejection treatment. Continuous monitoring of dd-cfDNA can rule out acute or chronic rejection [32].
4. Non-invasive detection of inadequate immunosuppression [38].
5. Absolute dd-cfDNA can differentiate between infection and rejection which was a limitation with fractional dd-cfDNA.
6. Cost-effectiveness surveillance of transplant recipients to reduce premature graft loss and re-transplantation.

#### 3.2.6. Disadvantages [51]

1. dd-cfDNA levels can increase during events other than rejection including BK nephritis, infections, and other causes of organ injury. It may not be reliable during these events.
2. Different tests have different thresholds for rejection and there is a need to understand the test characteristics.
3. Lack of data in long-term transplant and ongoing surveillance.
4. Lack of data in chronic rejection or subclinical rejection.
5. Not tested within 4 weeks of transplant.

### 3.3. Gene Expression Biomarkers

Gene expression profiling (GEP) is a sophisticated technique that can measure the transcriptional expression of numerous genes. In transplant recipients, these tests can identify disease processes like rejection. They utilize sophisticated technologies like DNA microarrays or gene sequencing, the former measures the activity of specific genes of interest and the latter measures all the active genes in a cell [52]. Genes related to immune cells (T cells, B cells, NK cells, interferon gamma, monocytes, macrophages), immunogenic pathways (interferon and interleukin signaling, neutrophil activation), and networks related to rejection are usually examined in GEP.

In transplantation, several studies have explored the utility of gene expression profiling (GEP) in predicting acute or subacute transplant rejection, chronic graft injury, immunosuppression optimization, and monitoring graft health [53,54,55].

Kurian et al. [53] performed whole gene expression profiling (GEP) using DNA microarrays in KT recipients in development and validation cohorts. In a data set of 148 peripheral blood samples using multiple three-way classifier tools, 2666 genes were significantly expressed. Among them, they identified the top 200 genes in diagnosing and classifying no rejection, AR, and acute dysfunction with no rejection (ADNR). The sensitivity, specificity, PPV, NPV, and AUC were 82% to 100%, 76% to 95%, 76% to 95%, 79% to 100%, 84% to 100%, and 0.817 to 0.968, respectively, The study concluded that peripheral blood gene expression profiling can be used as a minimally invasive tool to accurately reveal transplant excellence (TX), AR, and acute dysfunction with no rejection (ADNR) in the setting of acute kidney transplant dysfunction [53].

In a retrospective study, Banff Human Organ Transplant (B-HOT) NanoString gene panel was used in 340 renal biopsies (220 in discovery cohort and 129 in validation cohort). The primary purpose was to identify a set of genes on a kidney biopsy to more precisely diagnose antibody-mediated rejection (AMR). The study identified a nine-gene score that demonstrated higher accuracy of 0.92 for active AMR. This gene score showed strong correlation with histological AMR features on biopsy. The gene score was also found to independently predict risk of graft loss on multivariable analysis [56].

A multicenter prospective study (CTOT-08) [54] developed a 57-classifier gene panel to assess subclinical AR in early post-kidney-transplant patients with stable renal function. They initially conducted a discovery analysis followed by validation of their results. The test had a PPV of 47–61% and NPV of 78–88% in predicting subclinical AR. The test result is read as negative (transplant excellent or TX) or positive (non-TX) and is manufactured as TruGraf, Eurofins. The biomarker also independently correlated with BPAR, renal function, > grade 2 interstitial fibrosis, and de novo DSA formation. The results need to be validated in other prospective independent studies to assess their utility in clinical use.

The Kidney Solid Organ Response Test (kSORT, Immucor), is a quantitative-real-time-PCR-based 17-gene set developed to detect AR. In the initial study [55], the authors reported a high AUC of 0.95 in AR detection. Later, a large retrospective multicenter study with 1763 blood samples was unable to validate the kSORT assay in AR detection (AUC = 0.51) [57]. Currently, this test has been withdrawn from the market for further assessment and validation.

A smaller study [58] developed a 17-gene panel to identify subclinical AR at 3 months, future AR events, and risk of graft loss. This assay is being commercially developed (Tutevia, Verici) in prospective trials.

### 3.4. Combination Testing dd-cfDNA and GEP

The combination of dd-cfDNA and GEP represents a promising approach in transplant monitoring, offering complementary insights into allograft health. A significant study by Park et al. [59] performed a post hoc analysis on simultaneous use of dd-cfDNA and GEP (OmniGraf, Eurofins) in 428 samples (325 no rejection and 103 subclinical rejection) from 208 patients paired with surveillance biopsies at 2–6, 12, and 24 months post-transplant in the multicenter CTOT-08 study. Combined testing (GEP and dd-cfDNA) increased NPV to 88% and PPV to 81% compared to individual testing (NPV: 84% for cfDNA, 82% for GEP; PPV: 56% for dd-cfDNA, 47% for GEP) [59]. The combination testing may be be useful to distinguish TCMR (GEP) and AMR (dd-cfDNA) [60]. In April 2023, the CMS discontinued reimbursement of two simultaneous molecular tests. The manufacturer has withdrawn OmniGraf from the market since but it is currently marketed separately as TRAC (dd-cfDNA) and TruGraf (GEP).

### 3.5. Torque Teno Virus

Torque teno virus (TTV), discovered in 1997, is a small single-stranded DNA virus present in >90% of adults worldwide not known to cause disease [61]. It is classified in the genus *Alfatorquevirus* within the family Anelloviridae [62]. It is usually transmitted through various routes, including fecal–oral, parenteral, and vertical transmission from mother to child [63]. It can be detected in various fluids and tissues, but the highest concentration is found in bile [64], saliva, and feces. Just like polyomavirus, TTV levels can rise based on degree of net immunosuppression. As such, TTV levels are directly proportional to the intensity of immunosuppression, where high levels indicate overimmunosuppression and low levels indicate undersuppression and risk of rejection [65].

Studies on the role of TTV in predicting AR events are relatively sparse. In a meta-analysis [66] of seven studies, patients who developed AR had significantly lower TTV DNA load. The pooled sensitivity, specificity, and AUC in AR identification were 0.61, 0.81, and 0.79, respectively.

In the prospective TTV Quantification for the Prediction of Organ Rejection in Kidney Transplantation (TTV-POET) study [17], TTV levels were consecutively monitored in 386 consecutive KT recipients for 12 months per protocol. TTV load tends to peak at 3 months post-transplant and declines after 3 months. The odds of rejection decreased by 22% (OR 0.78) with each log increase in TTV load. Later a retrospective single-center study [67] validated these results. They identified decreased risk of rejection by 25% with every log_10_ increase in TTV load as quantified by CE commercial certified PCR (RR 0.75). Currently, TTV level has potential for development as a biomarker in quantifying the net degree of immunosuppression in SOT recipients, however, more studies need to be performed to assess its routine utility in transplant rejection [68].

### 3.6. Immunogenic Cells

The enzyme-linked immunosorbent spot (ELISPOT) assay was developed to assess interferon production from alloreactive T cells, providing insight into immune function after transplantation [69]. Mean frequencies of these T cells measured during the first 6 months post-transplantation correlated significantly with allograft function at both 6 and 12 months. Additionally, higher T-cell frequencies were observed in patients experiencing acute rejection. A subsequent study using a subset of CTOT-01 subjects confirmed these findings, particularly in individuals who did not receive induction therapy with rabbit antithymocyte globulin [70]. However, the usability of the ELISPOT assay remains limited due to conflicting results, variations in immunosuppressive protocols, ischemic times, and potential assay performance inconsistencies [71].

The Cylex ImmuKnow assay was developed to evaluate T-cell immune function by measuring intracellular adenosine triphosphate (ATP) levels. This assay helps identify patients at risk for either rejection or infection [72]. Lower ATP levels were associated with a higher degree of net level of immunosuppression, which increased the risk of infection, while higher ATP levels were linked to an elevated risk of rejection. The study found that patients with elevated ATP levels were at greater risk for rejection [72]. However, the test’s limitations include the need for serial measurements, variability based on timing after transplantation [73], and lack of any recent data.

Similarly, allospecific CD154+ T cells have been used to diagnose acute rejection (Pleximark^TM^), based on the immunoreactivity index (IR) which is a ratio of donor to third-party-induced response [74]. An IR >1 was suggestive of increased risk for rejection with a good sensitivity and specificity of almost 90% in diagnosing rejection. Another study included serial measurements and found similar results [75]. Limitations include the timing of the study, which was performed more than 1 year after transplantation, and small sample size.

### 3.7. Transitional B-Cell Cytokines

Cherukuri et al. [76] analyzed the ratio of interleukin-10 (IL-10) to tumor necrosis factor-α (TNFα) expressed by transitional B cells in detection of early TCMR. The authors found that the IL-10/TNFα ratio predicted clinical and subclinical rejection at 3 months after transplantation in three patient cohorts [76]. The IL-10/TNFα ratio offers another potential biomarker but external validation will be required before it can be implemented clinically.

### 3.8. Messenger-RNA-Based Signatures

Urinary messenger RNA (mRNA)-based signatures have been known to be predictive of AR and graft function in KT recipients. One of the biggest problems with mRNA signatures is their instability in the urine. Urine has mRNA-signature-degrading enzymes, necessitating immediate sample processing.

Suthanthiran et al. [77] describe three gene signature mRNAs (interferon-inducible protein 10 (IP-10) mRNA, 18S ribosomal RNA, and CD3 mRNA) to diagnose AR. This prospective multicenter CTOT-04 trial collected 4300 urine samples from 485 graft specimens from day 3 to 12 months post-transplant. Urinary pellets were prepared at each participating site center, stored at −80 °C, and shipped to a central laboratory. The signature had a sensitivity of 79%, specificity of 78%, and AUC of 0.85 in discriminating ACR from no rejection. It was also able to distinguish between ACR, AMR, and borderline rejection with an AUC of 0.78. The mRNA signature was able to predict rejection weeks prior to biopsy diagnosis of rejection. The same group [78] identified a five-gene mRNA signature in distinguishing AMR from TCMR with an AUC of 0.81 and a six-gene signature that distinguishes between AR and acute tubular injury with an AUC of 0.92.

Salinas et al. [79] developed a portable mRNA signature test to detect TCMR and BK virus nephropathy (BKVN). They utilized the three-gene assay from the CTOT-04 study for TCMR detection along with urinary cell BKV VP 1 mRNA copy number to detect BK nephropathy. The test was able to discriminate TCMR from the no TCMR/no BKVN group with an AUC of 0.84, sensitivity of 67%, and specificity of 86%. In this study, the authors developed a Zymo ZRC GF™ filter-based protocol that eliminated the complexity with centrifugation processes and storage at −80 °C. instead, KT recipients were trained to perform the initial filtration steps at home and store the sample at ambient temperatures to be shipped to the core laboratory for mRNA profiling. Since this study used a preselected data set, external validated studies are required to assess this test. This test is currently being developed as Uromap by Caredx.

### 3.9. Exosomes: Extracellular Vesicles

Exosomes are nanometer-sized extracellular vesicles (EVs) (between 50 and 200 nm) that are normally secreted by cells. They contain genetic material like messenger RNA (mRNA and microRNA (miRNA)), lipids, carbohydrates, and proteins like cytokines and chemokines [80]. They carry out cell-to-cell communication by releasing proteins and nucleic acids including mRNA and miRNA. Exosomes carry parent cell proteins and nucleic acids and therefore are reflective of a biologic function of the parent cell. In the transplanted kidney, exosomes are secreted from renal tubular cells, glomerular podocytes, and urothelium. EVs remain stable when secreted in urine unlike mRNA or proteins and can act as urinary biomarkers for KT rejection.

Park et al. [81] developed a urinary exosome assay to detect KT rejection called ‘integrated kidney exosome analysis’ (IKEA) using T-cell-derived CD3+ exosomes. CD3+ exosomes were significantly elevated in TCMR in both discovery and validation cohorts. IKEA had sensitivity, specificity, and AUC of 92.8%, 87.5%, and 0.84, respectively, in discriminating TCMR from no TCMR. The exosomal mRNA was stable in urine for 2 weeks when stored at 4 °C. The same group later identified and validated a 15-gene exosome mRNA signature to diagnose AR. This mRNA signature distinguished rejection and no rejection with an AUC of 0.93, NPV of 93.3%, and PPV of 86.2% [82]. The stability of EVs in the urine makes it a powerful biomarker for liquid biopsy.

### 3.10. Chemokines

Chemokines are chemotactic cytokines that guide directional movement of cells like leukocytes and endothelial and epithelial cells. The use of chemokines as biomarkers has been explored and validated in various independent studies [83]. Among them, T-cell chemoattractants, urinary chemokine 9 (CXCL9), and chemokine 10 (CXCL10) stand out as robust biomarkers that can be measured at the mRNA or protein level with high NPV in diagnosing rejection [84].

Kaminski et al. [85] explored the role of clustered regularly interspaced short palindromic repeats (CRISPR)/CRISPR-associated RNA targeted (CRISPR/Cas3) and Specific High-Sensitivity Enzymatic Reporter UnLOCKing (SHERLOCK) to detect BKV and cytomegalovirus and CXCL9 mRNA, a marker of acute rejection. CRISPR-based assay showed higher levels of CXCL9 in those with rejection with a sensitivity of 93% and ROC of 0.91 [85].

A single-center prospective cohort study [86] analyzed 1559 biopsy paired urinary samples from 622 KT recipients to assess the diagnostic performance of CXCL9 and CXCL10 integrated with clinical markers (estimated glomerular filtration rate (eGFR), DSA, polyoma viremia) in detecting AR. This integrated model was compared to histopathology. Chemokines logCXLC9/cr and logCXCL10/cr had ROC AUCs of 72 and 70, respectively. The combined performance of clinical markers (eGFR, proteinuria, HLA DSA) reached an ROC AUC of 75.9%. The integrated model combining chemokines with clinical markers had superior test characteristics with an ROC AUC of 81.3%, sensitivity of 75.2%, specificity of 71.4%, PPV of 22.2%, and NPV of 96.3% at the optimal threshold. The model has been validated in an external validation cohort which mimicked similar results [86].

The above data suggest that a clinical integrated model might provide superior value in detecting rejection and avoiding unnecessary biopsies. More prospective studies need to look at the role of such models before integrating into clinical practice.

### 3.11. Q-Score

Nolan et al. [87] developed a renal transplant Q-score from six Q-Sant biomarkers from urine (cell-free DNA, methylated-cell-free DNA, clusterin, CXCL10, creatinine, and urine total protein) to assess transplant health with a score ranging from 0–100. A Q-score of ≥32 was determined represent an increased risk of active rejection. At a prevalence of 25%, the Q-score cutoff of 32 had a sensitivity of 95.8%, specificity of 99.3%, NPV of 98.6%, and PPV of 98% in diagnosing active rejection. The Q-score was able to predict subclinical rejection 200 days before diagnosis on indication biopsy (sensitivity = 94.9%, specificity = 100%, and AUC = 0.99). The Q-score was also able to detect TCMR and AMR and differentiate them from stable allografts with an ROC of 99.8% and 98.2%, respectively [87]. Unfortunately, this assay has been withdrawn from the market for unknown reasons.

**Figure 1:**
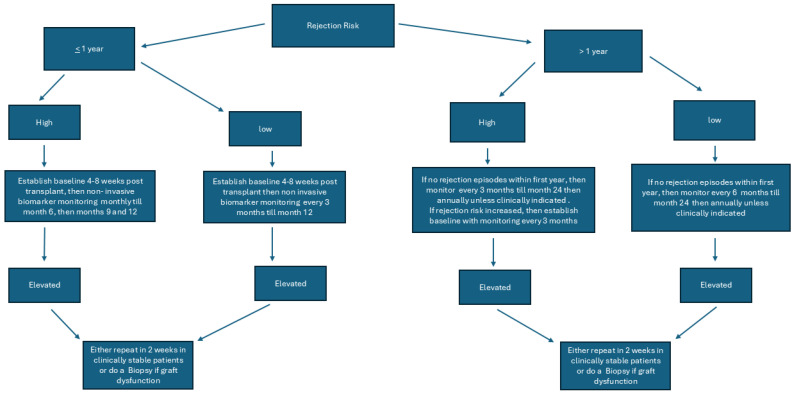
Pathway for proposed algorithm to utilize non-invasive biomarkers in kidney transplant recipients.

We recently published an editorial about the role of non-invasive biomarkers and some of the unanswered questions for future studies and readers are directed to review the same [88]. Our proposed algorithm for testing is Figure 1.

The proposed algorithm does not reflect when immunosuppression is decreased due to clinical indications like infection, GI illnesses, leukopenia, or for some other reason. Clinicians may use non-invasive biomarkers more frequently in such situations and frequency may be decided by the individual transplant center based on the patient’s risk for rejection and graft function.

## 4. Conclusions

The field of kidney transplant rejection monitoring is rapidly evolving, with significant advancements in the development of non-invasive biomarkers. Donor-derived cell-free DNA and gene expression profiling represent promising tools for detecting acute and subclinical rejection, offering advantages over traditional methods in terms of sensitivity, specificity, and patient safety. Emerging markers such as torque teno virus levels also show potential in guiding immunosuppression and identifying patients at risk for rejection. As research progresses, integrating these biomarkers into clinical practice may enhance early detection, personalize treatment strategies, and ultimately improve long-term allograft outcomes. Continued validation in diverse patient populations and standardized protocols will be essential to realizing their full clinical utility.

## Figures and Tables

**Table 1:** Summary of commercial donor-derived cell-free DNA assays available in the United States.

	AlloSure (CareDx)	Prospera (Natera)	TRAC (Viracor Eurofins)
Validation cohort	DART study (NCT02424227, 2015–2016) Prospective 14 sites 102 patients [15]	USCF biobank (pre-2018) ^a^ Retrospective 1 site 178 patients [14]	Undefined biobank (pre-2020) ^b^ 1 site 25 patients [44]
Diagnostic comparison	Histopathology	Histopathology	Histopathology
Suggested threshold for acute rejection	1%	1%	0.7%
Assay technology	Targeted next generation sequencing that uses 405 SNPs	Massively multiplexed PCR (mmPCR) and 13c,392 SNP	NGS targets 70,000 SNPs
Targeted diagnosis	Acute rejection (AMR > TCR)	Acute rejection (AMR > TCR)	Acute rejection (AMR > TCR)
Reported sensitivity and specificity	59%, 85%	89%, 73%	58%, 85%
NPV, PPV	84%, 61%	95%, 52%	86%, 55%
Potential false positive rate ^c^	15%	27%	15%
Turnaround time ^d^	2–3 days	3 days	4–5 days
Sample type	Whole blood	Whole blood	Whole blood
Commercial availability	FDA approved	FDA approved	Not yet approved

^a^ Study duration not included in landmark publication; ^b^ Initially studied in R&D setting, then subsequently studied post hoc via CTOT-08 (NCT01289717, 2011–2016) and single center biobank (Northwestern University); ^c^ At 25% prevalence; ^d^ Turnaround time may be subject to change.

## Data Availability

Not applicable for review article.

## Abbreviations

The following abbreviations are used in this manuscript

AAMR	Active antibody-mediated rejection
ADNR	Acute dysfunction with no rejection
AMR	Antibody-mediated rejection
AR	Acute rejection
ATP	Adenosine triphosphate
AUC	Receiver operating characteristic area under the curve
BL	Borderline
BKVN	BK virus nephropathy
CAAMR	Chronic active antibody-mediated rejection
cfDNA	Cell-free DNA
CMS	Center for Medicaid and Medicare Service
CRISPR	Clustered regularly interspaced short palindromic repeats
CXCL9	Chemokine 9
CXCL10	Chemokine 10
DART	Diagnosing acute rejection in kidney transplant recipients
dd-cfDNA	Donor-derived cell-free DNA
ddPCR	Digital droplet PCR
DSA	Donor-specific antibody
eGFR	Estimated glomerular filtration rate
ELISPOT	Enzyme-linked immunosorbent spot
ESRD	End stage renal disease
EV	Extracellular vesicle
GE	Genome equivalent
GEP	Gene expression profiling
HLA	Human leukocyte antigen
IL-10	Interleukin 10
IR	Immunoreactivity index
KSORT	Kidney solid organ response test
KT	Kidney transplant
KTRs	Kidney transplant recipients
LCD	Local coverage determination
miRNA	MicroRNA
mRNA	Messenger RNA
NGS	Next-generation sequencing
NLR	Negative likelihood ratio
NPV	Negative predictive value
OI	Other injury
OR	Odds ratio
PLR	Positive likelihood ratio
PPV	Positive predictive value
SNPs	Single-nucleotide polymorphisms
SOT	Solid organ transplant
STA	Stable allograft
TCMR	T-cell-mediated rejection
TNFα	Tumor necrosis factor alpha
TTV	Torque teno virus
TX	Transplant excellence

## References

[1] Sellares J., de Freitas D.G., Mengel M., Reeve J., Einecke G., Sis B., Hidalgo L.G., Famulski K., Matas A., Halloran P.F. (2012). Understanding the causes of kidney transplant failure: The dominant role of antibody-mediated rejection and nonadherence. Am. J. Transplant..

[2] Barker C.F., Markmann J.F. (2013). Historical overview of transplantation. Cold Spring Harb. Perspect. Med..

[3] OPTN/SRTR 2022 Annual Data Report. https://srtr.transplant.hrsa.gov/adr/adr2022.

[4] Hariharan S., Israni A.K., Danovitch G. (2021). Long-Term Survival after Kidney Transplantation. N. Engl. J. Med..

[5] Kaplan B., Meier-Kriesche H.U. (2002). Death after graft loss: An important late study endpoint in kidney transplantation. Am. J. Transplant..

[6] Muduma G., Odeyemi I., Smith-Palmer J., Pollock R.F. (2016). Review of the Clinical and Economic Burden of Antibody-Mediated Rejection in Renal Transplant Recipients. Adv. Ther..

[7] Labus A., Mucha K., Kulesza A., Fliszkiewicz M., Paczek L., Niemczyk M. (2022). Costs of Treatment of Acute Antibody-Mediated Rejection in Kidney Transplant Recipients. Transplant. Proc..

[8] Seifert M.E., Agarwal G., Bernard M., Kasik E., Raza S.S., Fatima H., Gaston R.S., Hauptfeld-Dolejsek V., Julian B.A., Kew C.E. (2021). Impact of Subclinical Borderline Inflammation on Kidney Transplant Outcomes. Transplant. Direct.

[9] Miller C.A., Fildes J.E., Ray S.G., Doran H., Yonan N., Williams S.G., Schmitt M. (2013). Non-invasive approaches for the diagnosis of acute cardiac allograft rejection. Heart.

[10] Schwarz A., Gwinner W., Hiss M., Radermacher J., Mengel M., Haller H. (2005). Safety and adequacy of renal transplant protocol biopsies. Am. J. Transplant..

[11] Naesens M., Anglicheau D. (2018). Precision Transplant Medicine: Biomarkers to the Rescue. J. Am. Soc. Nephrol..

[12] Desanti De Oliveira B., Xu K., Shen T.H., Callahan M., Kiryluk K., D’Agati V.D., Tatonetti N.P., Barasch J., Devarajan P. (2019). Molecular nephrology: Types of acute tubular injury. Nat. Rev. Nephrol..

[13] Quaglia M., Merlotti G., Guglielmetti G., Castellano G., Cantaluppi V. (2020). Recent Advances on Biomarkers of Early and Late Kidney Graft Dysfunction. Int. J. Mol. Sci..

[14] Sigdel T.K., Archila F.A., Constantin T., Prins S.A., Liberto J., Damm I., Towfighi P., Navarro S., Kirkizlar E., Demko Z.P. (2018). Optimizing Detection of Kidney Transplant Injury by Assessment of Donor-Derived Cell-Free DNA via Massively Multiplex PCR. J. Clin. Med..

[15] Bloom R.D., Bromberg J.S., Poggio E.D., Bunnapradist S., Langone A.J., Sood P., Matas A.J., Mehta S., Mannon R.B., Sharfuddin A. (2017). Cell-Free DNA and Active Rejection in Kidney Allografts. J. Am. Soc. Nephrol..

[16] Halloran P.F., Reeve J., Madill-Thomsen K.S., Demko Z., Prewett A., Gauthier P., Billings P., Lawrence C., Lowe D., Hidalgo L.G. (2023). Antibody-mediated Rejection Without Detectable Donor-specific Antibody Releases Donor-derived Cell-free DNA: Results From the Trifecta Study. Transplantation.

[17] Doberer K., Schiemann M., Strassl R., Haupenthal F., Dermuth F., Gorzer I., Eskandary F., Reindl-Schwaighofer R., Kikic Z., Puchhammer-Stockl E. (2020). Torque teno virus for risk stratification of graft rejection and infection in kidney transplant recipients-A prospective observational trial. Am. J. Transplant..

[18] Rutkowska J., Interewiczi B., Rydzewski A., Swietek M., Dominiak A., Durlik M., Olszewski W.L. (2007). Donor DNA is detected in recipient blood for years after kidney transplantation using sensitive forensic medicine methods. Ann. Transplant..

[19] Starzl T.E., Marchioro T.L., Waddell W.R. (1963). The Reversal of Rejection in Human Renal Homografts with Subsequent Development of Homograft Tolerance. Surg. Gynecol. Obstet..

[20] Starzl T.E., Demetris A.J., Murase N., Ildstad S., Ricordi C., Trucco M. (1992). Cell migration, chimerism, and graft acceptance. Lancet.

[21] Lo Y.M., Tein M.S., Pang C.C., Yeung C.K., Tong K.L., Hjelm N.M. (1998). Presence of donor-specific DNA in plasma of kidney and liver-transplant recipients. Lancet.

[22] Zhang J., Tong K.L., Li P.K., Chan A.Y., Yeung C.K., Pang C.C., Wong T.Y., Lee K.C., Lo Y.M. (1999). Presence of donor- and recipient-derived DNA in cell-free urine samples of renal transplantation recipients: Urinary DNA chimerism. Clin. Chem..

[23] Sigdel T.K., Vitalone M.J., Tran T.Q., Dai H., Hsieh S.C., Salvatierra O., Sarwal M.M. (2013). A rapid noninvasive assay for the detection of renal transplant injury. Transplantation.

[24] Alcaide M., Cheung M., Hillman J., Rassekh S.R., Deyell R.J., Batist G., Karsan A., Wyatt A.W., Johnson N., Scott D.W. (2020). Evaluating the quantity, quality and size distribution of cell-free DNA by multiplex droplet digital PCR. Sci. Rep..

[25] Sherwood K., Weimer E.T. (2018). Characteristics, properties, and potential applications of circulating cell-free dna in clinical diagnostics: A focus on transplantation. J. Immunol. Methods.

[26] Xu R.H., Wei W., Krawczyk M., Wang W., Luo H., Flagg K., Yi S., Shi W., Quan Q., Li K. (2017). Circulating tumour DNA methylation markers for diagnosis and prognosis of hepatocellular carcinoma. Nat. Mater..

[27] Chung D.C., Gray D.M., Singh H., Issaka R.B., Raymond V.M., Eagle C., Hu S., Chudova D.I., Talasaz A., Greenson J.K. (2024). A Cell-free DNA Blood-Based Test for Colorectal Cancer Screening. N. Engl. J. Med..

[28] Khan K.H., Cunningham D., Werner B., Vlachogiannis G., Spiteri I., Heide T., Mateos J.F., Vatsiou A., Lampis A., Damavandi M.D. (2018). Longitudinal Liquid Biopsy and Mathematical Modeling of Clonal Evolution Forecast Time to Treatment Failure in the PROSPECT-C Phase II Colorectal Cancer Clinical Trial. Cancer Discov..

[29] Norton M.E., Jacobsson B., Swamy G.K., Laurent L.C., Ranzini A.C., Brar H., Tomlinson M.W., Pereira L., Spitz J.L., Hollemon D. (2015). Cell-free DNA analysis for noninvasive examination of trisomy. N. Engl. J. Med..

[30] Beck J., Bierau S., Balzer S., Andag R., Kanzow P., Schmitz J., Gaedcke J., Moerer O., Slotta J.E., Walson P. (2013). Digital Droplet PCR for Rapid Quantification of Donor DNA in the Circulation of Transplant Recipients as a Potential Universal Biomarker of Graft Injury. Clin. Chem..

[31] Hindson C.M., Chevillet J.R., Briggs H.A., Gallichotte E.N., Ruf I.K., Hindson B.J., Vessella R.L., Tewari M. (2013). Absolute quantification by droplet digital PCR versus analog real-time PCR. Nat. Methods.

[32] Oellerich M., Sherwood K., Keown P., Schütz E., Beck J., Stegbauer J., Rump L.C., Walson P.D. (2021). Liquid biopsies: Donor-derived cell-free DNA for the detection of kidney allograft injury. Nat. Rev. Nephrol..

[33] Gielis E.M., Ledeganck K.J., Dendooven A., Meysman P., Beirnaert C., Laukens K., De Schrijver J., Van Laecke S., Van Biesen W., Emonds M.P. (2020). The use of plasma donor-derived, cell-free DNA to monitor acute rejection after kidney transplantation. Nephrol. Dial. Transplant..

[34] Lee H., Park Y.M., We Y.M., Han D.J., Seo J.W., Moon H., Lee Y.H., Kim Y.G., Moon J.Y., Lee S.H. (2017). Evaluation of Digital PCR as a Technique for Monitoring Acute Rejection in Kidney Transplantation. Genomics Inform..

[35] Xiao H., Gao F., Pang Q., Xia Q., Zeng X., Peng J., Fan L., Liu J., Wang Z., Li H. (2021). Diagnostic Accuracy of Donor-derived Cell-free DNA in Renal-allograft Rejection: A Meta-analysis. Transplantation.

[36] Xing Y., Guo Q., Wang C., Shi H., Zheng J., Jia Y., Li C., Hao C. (2024). Donor-derived cell-free DNA as a diagnostic marker for kidney-allograft rejection: A systematic review and meta-analysis. Biomol. Biomed..

[37] Wijtvliet V., Plaeke P., Abrams S., Hens N., Gielis E.M., Hellemans R., Massart A., Hesselink D.A., De Winter B.Y., Abramowicz D. (2020). Donor-derived cell-free DNA as a biomarker for rejection after kidney transplantation: A systematic review and meta-analysis. Transpl. Int..

[38] Oellerich M., Shipkova M., Asendorf T., Walson P.D., Schauerte V., Mettenmeyer N., Kabakchiev M., Hasche G., Grone H.J., Friede T. (2019). Absolute quantification of donor-derived cell-free DNA as a marker of rejection and graft injury in kidney transplantation: Results from a prospective observational study. Am. J. Transplant..

[39] Garg N. (2020). Donor-Derived Cell-Free DNA: Is It All the Same? The Jury Is Still Out. Kidney360.

[40] Pettersson L., Vezzi F., Vonlanthen S., Alwegren K., Hedrum A., Hauzenberger D. (2021). Development and performance of a next generation sequencing (NGS) assay for monitoring of mixed chimerism. Clin. Chim. Acta.

[41] Viracor TRAC Kidney dd-cfDNA Testing. https://www.eurofins-viracor.com/media/2q2l52ab/mm-0997-rev2-1219-trac-dd-cf-dna-kidney-lab-bulletin.pdf.

[42] Lum E.L., Nieves-Borrero K., Homkrailas P., Lee S., Danovitch G., Bunnapradist S. (2021). Single center experience comparing two clinically available donor derived cell free DNA tests and review of literature. Transplant. Rep..

[43] Melancon J.K., Khalil A., Lerman M.J. (2020). Donor-Derived Cell Free DNA: Is It All the Same?. Kidney360.

[44] Bixler E., Kleiboeker S.B. (2020). Donor-Derived Cell-Free DNA: Clinical Applications for the Diagnosis of Rejection.

[45] Huang E., Gillespie M., Ammerman N., Vo A., Lim K., Peng A., Najjar R., Sethi S., Jordan S.C., Mirocha J. (2020). Donor-derived Cell-free DNA Combined With Histology Improves Prediction of Estimated Glomerular Filtration Rate Over Time in Kidney Transplant Recipients Compared With Histology Alone. Transplant. Direct.

[46] Jordan S.C., Bunnapradist S., Bromberg J.S., Langone A.J., Hiller D., Yee J.P., Sninsky J.J., Woodward R.N., Matas A.J. (2018). Donor-derived Cell-free DNA Identifies Antibody-mediated Rejection in Donor Specific Antibody Positive Kidney Transplant Recipients. Transplant. Direct.

[47] Whitlam J.B., Ling L., Skene A., Kanellis J., Ierino F.L., Slater H.R., Bruno D.L., Power D.A. (2019). Diagnostic application of kidney allograft-derived absolute cell-free DNA levels during transplant dysfunction. Am. J. Transplant..

[48] Dauber E.-M., Kollmann D., Kozakowski N., Rasoul-Rockenschaub S., Soliman T., Berlakovich G.A., Mayr W.R. (2020). Quantitative PCR of INDELs to measure donor-derived cell-free DNA—A potential method to detect acute rejection in kidney transplantation: A pilot study. Transplant. Int..

[49] Kueht M.L., Dongur L.P., Cusick M., Stevenson H.L., Mujtaba M. (2022). The Current State of Donor-Derived Cell-Free DNA Use in Allograft Monitoring in Kidney Transplantation. J. Pers. Med..

[50] CMS (2024). MolDx Local Coverage Determination Statement. https://www.cms.gov/newsroom/press-releases/moldx-local-coverage-determination-statement.

[51] Chopra B., Sureshkumar K.K. (2021). Emerging role of cell-free DNA in kidney transplantation. World J. Exp. Med..

[52] Hurd P.J., Nelson C.J. (2009). Advantages of next-generation sequencing versus the microarray in epigenetic research. Brief. Funct. Genom. Proteomic.

[53] Kurian S.M., Williams A.N., Gelbart T., Campbell D., Mondala T.S., Head S.R., Horvath S., Gaber L., Thompson R., Whisenant T. (2014). Molecular classifiers for acute kidney transplant rejection in peripheral blood by whole genome gene expression profiling. Am. J. Transplant..

[54] Friedewald J.J., Kurian S.M., Heilman R.L., Whisenant T.C., Poggio E.D., Marsh C., Baliga P., Odim J., Brown M.M., Ikle D.N. (2019). Development and clinical validity of a novel blood-based molecular biomarker for subclinical acute rejection following kidney transplant. Am. J. Transplant..

[55] Roedder S., Sigdel T., Salomonis N., Hsieh S., Dai H., Bestard O., Metes D., Zeevi A., Gritsch A., Cheeseman J. (2014). The kSORT assay to detect renal transplant patients at high risk for acute rejection: Results of the multicenter AART study. PLoS Med..

[56] Beadle J., Papadaki A., Toulza F., Santos E., Willicombe M., McLean A., Peters J., Roufosse C. (2023). Application of the Banff Human Organ Transplant Panel to kidney transplant biopsies with features suspicious for antibody-mediated rejection. Kidney Int..

[57] Van Loon E., Giral M., Anglicheau D., Lerut E., Dubois V., Rabeyrin M., Brouard S., Roedder S., Spigarelli M.G., Rabant M. (2021). Diagnostic performance of kSORT, a blood-based mRNA assay for noninvasive detection of rejection after kidney transplantation: A retrospective multicenter cohort study. Am. J. Transplant..

[58] Zhang W., Yi Z., Keung K.L., Shang H., Wei C., Cravedi P., Sun Z., Xi C., Woytovich C., Farouk S. (2019). A Peripheral Blood Gene Expression Signature to Diagnose Subclinical Acute Rejection. J. Am. Soc. Nephrol..

[59] Park S., Guo K., Heilman R.L., Poggio E.D., Taber D.J., Marsh C.L., Kurian S.M., Kleiboeker S., Weems J., Holman J. (2021). Combining Blood Gene Expression and Cellfree DNA to Diagnose Subclinical Rejection in Kidney Transplant Recipients. Clin. J. Am. Soc. Nephrol..

[60] Heilman R.L., Fleming J.N., Park S.H., Rebello C., Kleiboeker S., Holman J., Friedewald J.J. (2024). Clinical Value of Peripheral Blood Gene Expression Profile and dd-cfDNA for Identifying Persistent Rejection. Kidney360.

[61] Kuczaj A., Przybylowski P., Hrapkowicz T. (2023). Torque Teno Virus (TTV)-A Potential Marker of Immunocompetence in Solid Organ Recipients. Viruses.

[62] ICTV Anelloviridae. https://ictv.global/report_9th/ssDNA/Anelloviridae.

[63] Rezahosseini O., Drabe C.H., Sorensen S.S., Rasmussen A., Perch M., Ostrowski S.R., Nielsen S.D. (2019). Torque-Teno virus viral load as a potential endogenous marker of immune function in solid organ transplantation. Transplant. Rev..

[64] Reshetnyak V.I., Maev I.V., Burmistrov A.I., Chekmazov I.A., Karlovich T.I. (2020). Torque teno virus in liver diseases: On the way towards unity of view. World J. Gastroenterol..

[65] Gore E.J., Gard L., Niesters H.G.M., Van Leer Buter C.C. (2023). Understanding torquetenovirus (TTV) as an immune marker. Front. Med..

[66] Zeng J., Tang Y., Lin T., Song T. (2023). Torque-teno virus for the prediction of graft rejection and infection disease after kidney transplantation: A systematic review and meta-analysis. J. Med. Virol..

[67] Gorzer I., Haupenthal F., Maggi F., Gelas F., Kulifaj D., Brossault L., Puchhammer-Stockl E., Bond G. (2023). Validation of plasma Torque Teno viral load applying a CE-certified PCR for risk stratification of rejection and infection post kidney transplantation. J. Clin. Virol..

[68] Torque Teno Virus: A Biomarker of Immunosuppression. https://clinicaltrials.gov/study/NCT05756036?term=NCT05756036&rank=1.

[69] Hricik D.E., Rodriguez V., Riley J., Bryan K., Tary-Lehmann M., Greenspan N., Dejelo C., Schulak J.A., Heeger P.S. (2003). Enzyme linked immunosorbent spot (ELISPOT) assay for interferon-gamma independently predicts renal function in kidney transplant recipients. Am. J. Transplant..

[70] Hricik D.E., Augustine J., Nickerson P., Formica R.N., Poggio E.D., Rush D., Newell K.A., Goebel J., Gibson I.W., Fairchild R.L. (2015). Interferon Gamma ELISPOT Testing as a Risk-Stratifying Biomarker for Kidney Transplant Injury: Results From the CTOT-01 Multicenter Study. Am. J. Transplant..

[71] Girmanova E., Hruba P., Viklicky O., Slavcev A. (2022). ELISpot assay and prediction of organ transplant rejection. Int. J. Immunogenet..

[72] He J., Li Y., Zhang H., Wei X., Zheng H., Xu C., Bao X., Yuan X., Hou J. (2013). Immune function assay (ImmuKnow) as a predictor of allograft rejection and infection in kidney transplantation. Clin. Transplant..

[73] Gralla J., Huskey J., Wiseman A.C. (2012). Trends in immune function assay (ImmuKnow; Cylex) results in the first year post-transplant and relationship to BK virus infection. Nephrol. Dial. Transplant..

[74] Ashokkumar C., Shapiro R., Tan H., Ningappa M., Elinoff B., Fedorek S., Sun Q., Higgs B.W., Randhawa P., Humar A. (2011). Allospecific CD154+ T-cytotoxic memory cells identify recipients experiencing acute cellular rejection after renal transplantation. Transplantation.

[75] Rohan V.S., Soliman K.M., Alqassieh A., Alkhader D., Patel N., Nadig S.N. (2020). Renal allograft surveillance with allospecific T-cytotoxic memory cells. Ren. Fail..

[76] Cherukuri A., Salama A.D., Mehta R., Mohib K., Zheng L., Magee C., Harber M., Stauss H., Baker R.J., Tevar A. (2021). Transitional B cell cytokines predict renal allograft outcomes. Sci. Transl. Med..

[77] Suthanthiran M., Schwartz J.E., Ding R., Abecassis M., Dadhania D., Samstein B., Knechtle S.J., Friedewald J., Becker Y.T., Sharma V.K. (2013). Urinary-cell mRNA profile and acute cellular rejection in kidney allografts. N. Engl. J. Med..

[78] Matignon M., Ding R., Dadhania D.M., Mueller F.B., Hartono C., Snopkowski C., Li C., Lee J.R., Sjoberg D., Seshan S.V. (2014). Urinary cell mRNA profiles and differential diagnosis of acute kidney graft dysfunction. J. Am. Soc. Nephrol..

[79] Salinas T., Li C., Snopkowski C., Stryjniak G., Shankaranarayanan D., Albakry S., Ding R., Sharma V.K., Salvatore S.P., Seshan S.V. (2023). Urinary cell mRNA profiling of kidney allograft recipients: Development of a portable protocol for noninvasive diagnosis of T cell mediated rejection and BK virus nephropathy. J. Immunol. Methods.

[80] Ramalhete L., Araujo R., Ferreira A., Calado C.R.C. (2024). Exosomes and microvesicles in kidney transplantation: The long road from trash to gold. Pathology.

[81] Park J., Lin H.Y., Assaker J.P., Jeong S., Huang C.H., Kurdi T., Lee K., Fraser K., Min C., Eskandari S. (2017). Integrated Kidney Exosome Analysis for the Detection of Kidney Transplant Rejection. ACS Nano.

[82] El Fekih R., Hurley J., Tadigotla V., Alghamdi A., Srivastava A., Coticchia C., Choi J., Allos H., Yatim K., Alhaddad J. (2021). Discovery and Validation of a Urinary Exosome mRNA Signature for the Diagnosis of Human Kidney Transplant Rejection. J. Am. Soc. Nephrol..

[83] Hu H., Aizenstein B.D., Puchalski A., Burmania J.A., Hamawy M.M., Knechtle S.J. (2004). Elevation of CXCR3-binding chemokines in urine indicates acute renal-allograft dysfunction. Am. J. Transplant..

[84] Rabant M., Amrouche L., Morin L., Bonifay R., Lebreton X., Aouni L., Benon A., Sauvaget V., Le Vaillant L., Aulagnon F. (2016). Early Low Urinary CXCL9 and CXCL10 Might Predict Immunological Quiescence in Clinically and Histologically Stable Kidney Recipients. Am. J. Transplant..

[85] Kaminski M.M., Alcantar M.A., Lape I.T., Greensmith R., Huske A.C., Valeri J.A., Marty F.M., Klambt V., Azzi J., Akalin E. (2020). A CRISPR-based assay for the detection of opportunistic infections post-transplantation and for the monitoring of transplant rejection. Nat. Biomed. Eng..

[86] Van Loon E., Tinel C., de Loor H., Bossuyt X., Callemeyn J., Coemans M., De Vusser K., Sauvaget V., Olivre J., Koshy P. (2024). Automated Urinary Chemokine Assays for Noninvasive Detection of Kidney Transplant Rejection: A Prospective Cohort Study. Am. J. Kidney Dis..

[87] Nolan N., Valdivieso K., Mani R., Yang J.Y.C., Sarwal R.D., Katzenbach P., Chalasani K., Hongo D., Lugtu G., Mark C. (2020). Clinical and Analytical Validation of a Novel Urine-Based Test for the Detection of Allograft Rejection in Renal Transplant Patients. J. Clin. Med..

[88] Mour G., Parajuli S. (2024). Can Blood Gene Expression Profile and Donor-Derived Cellfree DNA Guide Postrejection Management among Kidney Transplant Recipients?. Kidney360.

